# Comparative analysis and innovation of a simple and rapid method for high-quality RNA and DNA extraction of kiwifruit

**DOI:** 10.1016/j.mex.2018.03.008

**Published:** 2018-04-10

**Authors:** Mansour Afshar-Mohammadian, Mohammad Hossein Rezadoost, Seyyed Fatemeh Fallah

**Affiliations:** a Department of Biology, Faculty of Sciences, University of Guilan, Iran; b Department of Biotechnology, Faculty of Agricultural Sciences, University of Guilan, Iran

**Keywords:** RNA and DNA extraction for fleshy fruits, RNA and DNA extraction, RT-qPCR, Kiwifruit, Polyphenols, Polysaccharides

## Abstract

RNA and DNA extraction is a requirement for the study of gene expression and has an increasingly important role in genetic studies of all fleshy fruits. RNA and DNA extraction is difficult in kiwifruit due to the significant amount of polysaccharides and polyphenols compounds. So far, no commercial kit has been developed specifically for high-quality RNA and DNA extraction in kiwifruit and the common protocols for RNA extraction have poor yields. This study developed a new protocol for high quality RNA extraction in *Actinidia deliciosa*. According to the results, the average yield of RNA extraction of fruit and leaf of *A. deliciosa* was ∼2180.7 ng/μl (∼545.175 μg/g FW) and ∼3424.9 ng/μl (∼856.225 μg/g FW), respectively with A_260_/A_280_ between 1.95 to 2.07 and A_260_/A_230_ higher than 2 indicating high RNA purity. While the averages yield of RNA extraction using previous methods from kiwifruit and leaf was 23 μg/g FW and 527 μg/g FW, respectively. Also, the average yields of genomic DNA from kiwifruit ranged from 52 to 98 ng/μl with A_260_/A_230_ between 0.60 to 1.64 and A_260_/A_280_ between 1.40 to 1.48. To our knowledge, this is the first report of a highly efficient and rapid method of RNA and DNA extraction in kiwifruit which can be used for a broad spectrum of the all fleshy fruits.

## Graphical abstract

**Figure:**
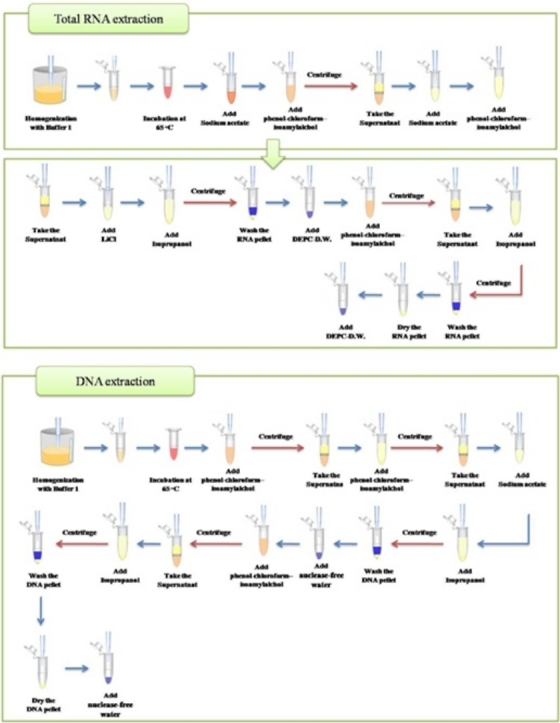

## Method details

### Preparation of material

Hayward kiwifruit samples from the cultivar of *A. deliciosa* with a mean soluble solid content of 6.1–6.95% and firmness of 80–100 N were collected from the research center gardens close to the city of Tonekabon at the north of Iran, at early November 2016. The samples for RNA extraction comprised outer pericarp (the green part, without seeds) were immediately frozen in liquid nitrogen and stored at −80 °C until the RNA was extracted.

### Reagent preparation

#### Buffers

**Extraction Buffer 1**: 200 mM Tris–HCl (pH 8), 1.4 M NaCl, 25 mM EDTA, 3% (w/v) CTAB, Add β-mercaptoethanol to a final concentration of 2% (v/v) just before use.

**Extraction Buffer 2**: RNX-Plus (Cinnagen, RNX-Plus is a Guanidine/phenol solution for total RNA isolation from homogenized sample).

**Extraction Buffer 3**: Buffer RLT and Buffer RLC (RNeasy Plant Mini Kit, Qiagen).

#### Reagents

2 M Sodium acetate (2 M sodium acetate preparation for RNA extraction: add 16.42 g sodium acetate (anhydrous) to 40 ml water and 35 ml glacial acetic acid. Adjust to a pH of 4 with glacial acetic acid and bring to a final volume of 100 ml with DEPC-treated water), 2 M LiCl, chloroform–isoamylalcohol (24:1, v/v), phenol-chloroform–isoamylalchol (25:24:1, v/v), 4 M NaCl, 2 M guanidinethiocyanate, polyvinylpolypyrrolidone (PVPP), isopropanol, 70% (v/v) ethanol (EtOH).

### Regular procedure

#### RNA extraction protocol

1 Scrap 200 mg of fruit tissue in a 2-ml tube.
2 Add 1 ml extraction buffer 1, 4% (w/v) PVPP and 2% (v/v) β-mercaptoethanol to sample tissue and were ground to TissuRuptor, then vortex for 20 s and transfer the tube to the heat sink at 65 °C for 15 min.
3 Then, place the tube on ice for 5 min and add 1/4 vol 2 M sodium acetate.
4 Place the tube on ice for 5 min and add 500 μl phenol chloroform–isoamylalchol (25:24:1, v/v), then vortex for 1 min and centrifuge at 12,000 rpm for 15 min at 4 °C.
5 Transfer 700 μl of supernatant to a new tube and add 1/4 vol 2 M sodium acetate and place the tube on ice for 5 min
6 Add 500 μl phenol chloroform–isoamylalchol (25:24:1, v/v), then vortex for 1 min and centrifuge at 14,000 rpm for 5 min at 4 °C.
7 Transfer 700 μl of supernatant to a new tube and add 1/2 of the total volume 2 M LiCl and keep for 20 min on ice.
8 Add 900 μl isopropanol and store for 1 h at −20 C and Centrifuge at 14,000 rpm at 4 °C for 20 min (in this stage the pellet should be seen).
9 Wash the pellet with 70% ethanol (add ethanol gently and keep for 2 min at room temperature, do not spin, be careful that the pellets do not spill out then centrifuge at 8000 rpm for 2 min).
10 Add 300 μl DEPC-treated water, then add 500 μl phenol-chloroform-isoamylalchol (25:24:1, v/v) and centrifuge at 13,000 rpm for 10 min at 4 °C.
11 Supernatant to a new tube and add 1 ml isopropanol and place the tube on ice for 5 min and centrifuge at 14,000 rpm at 4 °C for 20 min.
12 Subsequently, wash the pellet with 70% ethanol (add ethanol gently and keep for 2 min at room temperature, do not spin, be careful that the pellets do not spill out then centrifuge at 8000 rpm for 2 min).
13 Finally, pellet should be dried and dissolved in 50 μl DEPC-treated water and stored at −80 °C.

#### DNA extraction protocol

1 Scrap 200 mg of fruit tissue in a 2-ml tube.
2 Add 1 ml extraction buffer 1, 4% (w/v) PVPP and 2% (v/v) β-mercaptoethanol to sample tissue and ground to TissuRuptor, then vortex for 20 s and transfer the tube to the heat sink at 65 °C for 15 min.
3 Place the tube on ice for 10 min and add 500 μl phenol chloroform–isoamylalchol (25:24:1, v/v), then vortex for 1 min and centrifuge at 12,000 rpm for 15 min at 4 °C.
4 Transfer 700 μl of supernatant to a new tube and add 500 μl phenol chloroform–isoamylalchol (25:24:1, v/v), then vortex for 1 min and centrifuge at 10,000 rpm for 5 min at 4 °C.
5 Transfer 700 μl of supernatant to a new tube and add 1/2 vol of supernatant, 2 M sodium acetate.
6 Place the tube on ice for 15 min and Add 800 μl isopropanol and place the tube on ice for 30 min, then centrifuge at 12,000 rpm for 20 min at 4 °C.
7 Wash the pellet with 70% ethanol (add ethanol gently and keep for 2 min at room temperature, do not spin, be careful that the pellets do not spill out then centrifuge at 8000 rpm for 2 min).
8 Add 300 μl nuclease-free water, then add 500 μl phenol-chloroform–isoamylalchol (25:24:1, v/v) and centrifuge at 13,000 rpm for 10 min at 4 °C.
9 Then, add 500 μl isopropanol and place the tube on ice for 5 min and centrifuge at 14,000 rpm at 4 °C for 10 min.
10 Subsequently, wash the pellet with 70% ethanol (add ethanol gently and keep for 2 min at room temperature, do not spin, be careful that the pellets do not spill out then centrifuge at 8000 rpm for 2 min).
11 Finally, pellet was dried and dissolved in 50 μl nuclease-free water and stored at −80 °C.

### Modified procedure

This modified procedure contains steps which are mentioned in the above procedure (regular procedure).

#### RNA extraction protocol

##### Protocol 1

It was similar to new protocol (regular procedure), with this difference that the centrifugation process was at low speeds (10,000 rpm at stage 4 and 12,000 rpm at step 6).

##### Protocol 2

It was similar to new protocol, with this difference that after step 3 and before step 4, guanidinethiocyanate was used (add 300 μl 2 M guanidinethiocyanate and place the tube on ice for 5 min).

##### Protocol 3

It was similar to new protocol, with this difference that chloroform–isoamylalchol (24:1, v/v) was used instead of phenol chloroform–isoamylalchol (25:24:1, v/v) without the steps of 9, 10 and 11.

##### Protocol 4

1 Scrap 200 mg of fruit tissue in a 2-ml tube.
2 Add 1 ml extraction buffer 1, 4% (w/v) PVPP and 2% (v/v) β-mercaptoethanol sample tissue and were ground to TissuRuptor, then vortex for 20 s and transfer the tube to the heat sink at 65 °C for 15 min.
3 Place the tube on ice for 5 min and add 1/2 vol 2 M sodium acetate.
4 Later the mixture, place the tube on ice for 5 min and add 500 μl chloroform–isoamylalchol (24:1, v/v), then vortex for 1 min and centrifuged at 14,000 rpm for 15 min at 4 °C.
5 Transfer 700 μl of supernatant to a new tube and add 1/2 of the total volume 2 M LiCl and keep for 20 min on ice.
6 Then, add 900 μl isopropanol and store for 1 h at −20 °C and Centrifuge at 14,000 rpm at 4 °C for 20 min (in this stage the pellet should be seen).
7 Subsequently, wash the pellet with 70% ethanol (add ethanol gently and keep for 2 min at room temperature, do not spin, be careful that the pellets do not spill out then centrifuge at 8000 rpm for 2 min).
8 Finally, pellet should be dried and dissolved in 50 μl DEPC-treated water and stored at −80 °C.

##### Protocol 5

1 Scrap 200 mg of fruit tissue in a 2-ml tube.
2 Add 1 ml extraction buffer 1, 4% (w/v) PVPP and 2% (v/v) β-mercaptoethanol to sample tissue and were ground to TissuRuptor, then vortex for 20 s and transfer the tube to the heat sink at 65 °C for 15 min.
3 Place the tube on ice for 10 min and add 1/4 vol 2 M sodium acetate.
4 Later the mixture, place the tube on ice for 5 min and add 500 μl chloroform–isoamylalchol (24:1, v/v), then vortex for 1 min and centrifuged at 12,000 rpm for 15 min at 4 °C.
5 Transfer 450 μl of supernatant to a new tube and add 1/4 vol 2 M sodium acetate.
6 Place the tube on ice for 5 min and add 500 μl chloroform–isoamylalchol (24:1, v/v), then vortex for 1 min and centrifuged at 13,000 rpm for 10 min at 4 °C.
7 Transfer 500 μl of supernatant to a new tube and add 500 μl isopropanol and store for 0.5 h at room temperature and centrifuge at 14,000 rpm at 4 °C for 20 min (in this stage the pellet should be seen).
8 Subsequently, wash the pellet with 70% ethanol (add ethanol gently and keep for 2 min at room temperature, do not spin, be careful that the pellets do not spill out then centrifuge at 8000 rpm for 2 min).
9 Finally, pellet should be dried and dissolved in 50 μl DEPC-treated water and stored at −80 °C.

##### Protocol 6

It was similar to protocol 5 with this difference that 1/4 vol 4 M NaCl was used instead of 1/4 vol 2 M sodium acetate at step 3 and 5.

##### Protocol 7

It was similar to protocol 5, with this difference that buffer 2 (RNX-Plus) was used instead of buffer 1 and also after step 6, transfer 500 μl of supernatant to a new tube and add 1/2 of the total volume 2 M LiCl and keep for 20 min on ice.

##### Protocol 8

It was similar to protocol 6, with this difference that buffer 2 (RNX-Plus) was used instead of buffer 1 and after step 6, transfer 500 μl of supernatant to a new tube and add 1/2 of the total volume 2 M LiCl and keep for 20 min on ice.

##### Protocol 9

It was similar to protocol 8, with this difference that buffer 1 was used and without step 5.

##### Protocol 10

Extraction method using an RNX-Plus kit according to the manufacturer’s protocol.

##### Protocol 11

Extraction method using an RNeasy Plant Mini Kit (Qiagen) according to the manufacturer’s protocol.

#### DNA extraction protocol

##### Protocol 1

It was similar to method 9, with the difference that steps of 8, 9 and 10 were not used.

##### RNA and DNA yields, quality and RT-qPCR conditions

The concentration and purity of the extracted RNA and DNA samples were quantified with NanoDrop^®^ (Termo One C). The absorption ratios A_260_/A_230_ and A_260_/A_280_ were used to detect polysaccharide/polyphenolic contaminants and protein contaminants, respectively. Integrity of the RNA samples was assessed on a 1% denaturing formaldehyde agarose gels by electrophoresis at 50 V [1].

Total RNA was extracted from outer pericarp tissue and treated with DNase I (RNase Free, Cinnagen) was used for removing contaminating DNA. After DNase treatment, the cDNA was synthesized from 2.5 μg of DNA-free RNA with a cDNA Synthesis kit (Fermentas) following the manufacturer’s protocol with Superscript III (Invitrogen), and oligo d(T)20 to a total volume of 20 μg. The synthesized cDNA was used in a reaction for PCR in order to estimate the expression level of the actin gene. Kiwifruit actin was used as an internal control to normalize small differences in template amounts with the forward primer (5’-GGAAGCTGCAGGAATCCATG-3’) and reverse primer (5’-CCTCCAATCCAGACGCTGTA-3’). The following PCR program was used: 95 °C for 5 min, 40 cycles of 94 °C for 30 s, 60 °C for 30 s, and 72 °C for 30 s. The program ended with a 10 min extension at 72 °C. The amplified products were separated on a 1% agarose/TBE gel electrophoresis and imagined after staining with Loading Dye ((Buffer) 6x Cinnagen [2,3].

## Method validation

Some protocols such as those described by Smart and Roden [1], Minguzzi et al. [4], Ma et al. [5], and Yockteng et al. [6] which are used for RNA extraction from plant tissues had low yield because of the high amount of polyphenols, polysaccharides and other secondary metabolites in the plant tissues. Meanwhile, most of the examined isolation methods were complicated. By the way, the extraction techniques and the quality of the products, the relative analysis of genomes or transcriptomes across plant and tissue types bring a challenge for researchers. The majority of the protocols are not completely satisfying as they may be time using up, technically complicated, need ultracentrifugation steps and are specific to a particular plant species.

To our knowledge, this is the first report of a highly efficient method for RNA and DNA extraction from kiwifruit.

**Fig. 1:**
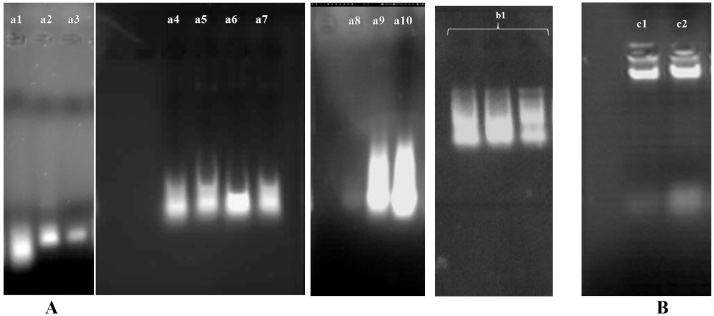
Comparison of total RNA isolated (A) and DNA isolated (B) from protocols of *A. deliciosa*. a1–a11: Kiwifruit RNA, b1: leaves RNA and c1-c2: Kiwifruit DNA. a1:protocol 9, a2: protocol 8, a3: protocol 7, a4: protocol 6, a5: protocol 5, a6: protocol 4, a7: protocol 3, a8: protocol 2, a9: protocol 1, a10: new protocol (Regular Procedure), b1: new protocol (Regular Procedure) in leaves, c1: Protocol 1 and c2: new Protocol (Regular Procedure).

Purity and concentration of RNA and DNA, extracted by different examined methods came in Table 1, Table 2. The results showed that the average yields of total RNA from fruit and leaf of *A. deliciosa* was ∼2180.7 ng/μl (∼545.175 μg/g FW) and ∼3424.9 ng/μl (∼856.225 μg/g FW), respectively. Absorption of A_260_/A_230_ and A_260_/A_280_ ratios in our new protocol (Regular Procedure) was higher than 2.0 using indicating the high purity of RNA samples without polyphenols and polysaccharides contamination. Also, the high absorption of A_260_/A_280_ ratios indicates a very low proteins contamination. On the other hand, the average absorption of A_260_/A_230_ of RNA examined using other protocols ranged from 0.15 to 2.02 and the average absorption of A_260_/A_280_ from 1.33 to 2.0 indicating the samples contamination by polyphenols, polysaccharides and proteins. Nevertheless, the average RNA yields using other protocols were far less and ranged from 28.09 to 14,445.6 ng/μl. The RNA extracted by new protocol, protocols 1 and 2 of *A. deliciosa* shown in Fig. 1, respectively, when separated on a 1% agarose/TBE gel electrophoresis. The RNA extracted by the protocol 2 (Fig. 1A) using 2 M guanidinethiocyanate for RNA sample did not show clear and intense bands. In comparison between new protocols and protocol 1, the new protocol with centrifugation steps at high speed of 14,000 rpm gave a better absorptions ratios (A_260_/A_230_ and A_260_/A_280_) and RNA yield than the protocol 1. Also, comparing the examined methods showed that using Re-extraction of 2 M sodium acetate and phenol-chloroform–isoamylalchol and 2 M LiCl (25:24:1, v/v) gave a better absorption ratios (A_260_/A_230_ and A_260_/A_280_) and RNA yield than using chloroform–isoamylalchol (24:1, v/v), 4 M NaCl without 2 M LiCl.

**Table 1:** Yield and purity of total RNA prepared by the new protocol and other protocols evaluated by NanoDrop^®^ (Termo One C) and ratios of A_260_/A_280_ and A_260_/A_230_.

Protocols Tissue		RNA Yield^a^ (ng/μL)	Absorbance ratio	
			A_260_/A_280_	A_260_/A_230_
New Protocol	Fruit	2180.7	2.07	2.43
(Regular Procedure)	Leaf	3424.9	1.95	2.31
Protocol 1	Fruit	976.1	1.58	2.02
Protocol 2	Fruit	14,445.6	1.33	2.02
Protocol 3	Fruit	232.3	2.00	1.57
Protocol 4	Fruit	283.1	1.93	1.46
Protocol 5	Fruit	212.0	1.83	1.06
Protocol 6	Fruit	135.8	1.89	1.14
Protocol 7	Fruit	87.9	1.69	0.27
Protocol 8	Fruit	54.5	1.74	0.15
Protocol 9	Fruit	162.7	1.90	1.05
RNX-Plus	Fruit	28.9	2.05	1.99
RNeasy Plant Mini Kit (Qiagen)	Fruit	38.5	1.45	0.46

a Results are expressed as the mean of 3 samples.

**Table 2:** Yield and purity of genomic DNA prepared by the new protocol evaluated by NanoDrop^®^ (Termo One C) and ratios of A_260_/A_280_ and A_260_/A_230_.

Protocols Tissue	DNA Yield^a^ (ng/μL)	Absorbance ratio	
		A_260_/A_280_	A_260_/A_230_
New protocol fruit (Regular Procedure)	98	1.48	1.64
protocol 1 fruit	52	1.40	0.60

a Results are expressed as the mean of 3 samples.

Also, the average yields of genomic DNA from kiwifruit ranged from 52 to 98 ng/μl with A_260_/A_230_ between 0.60 to 1.64 and A_260_/A_280_ between 1.40 to 1.48. Absorption of A_260_/A_230_ and A_260_/A_280_ ratios in the new protocol (Regular Procedure) was better than protocol 1 indicating the high purity of DNA samples and low polyphenols and polysaccharides contamination. The genomic DNA using new protocol and protocol 1 of *A. deliciosa* when separated on a 1% agarose/TBE gel electrophoresis, shown in Fig. 1B, respectively.

Proteins, lipids, carbohydrates, and cell debris are eliminated through extraction of the aqueous phase with the organic mixture of phenol and chloroform. Re-extraction of the aqueous phase along with the sodium acetate and phenol-chloroform-isoamylalchol reduced the polysaccharide and protein contamination [7,8]. The most important role of sodium acetate is binding the ions with nucleic acid. So that, in acidic condition (pH: 4) total RNA will remain in the upper aqueous phase of the whole mixture, while DNA and proteins remain in the inter phase or lower organic phase [9]. The most important role of LiCl is precipitating RNA, which is due to the relatively specific tendency to bind with RNA instead of DNA and removed phenol contamination. High LiCl concentrations may lead to an increase the contaminations (polysaccharides and polyphenols) in RNA extractions. RNA was precipitated using cold absolute ethanol/isopropanol to avoid any water insoluble precipitation and loss of RNA [10].

In the protocols 7 and 8 for RNA extraction, using buffer 1 (CTAB buffer) and buffer 2 (RNX-Plus) showed that buffer 1 did show higher RNA yield compared to the yield obtained with buffer 2. Meanwhile, the samples could not be imagined on a 1% agarose/TBE gel electrophoresis because RNA yield and absorptions were very low. Based on the results obtained from RNA extracted using RNeasy Plant Mini (Qiagen) Kit produced higher yield as compared with RNX-PLUS Kit using two different lysis buffers. However, it had very low RNA purity and so no bands could be observed on the 1% agarose/TBE gel electrophoresis. Nevertheless, both kits showed low concentration of RNA. While, the protocol 1 produced a more stable and higher yield of RNA extraction of *A. deliciosa* compared to the mentioned commercial kits.

In the current study, CTAB buffer was used for the extraction of RNA. Typically, the manual method of RNA isolation involves the usage of CTAB, SDS, phenol and high molarity guanidium salts [11]. Cetyltrimethylammonium bromide (CTAB) is a nonionic detersive that can precipitate nucleic acids and acidic polysaccharides from low ionic vigor solutions. Furthermore, proteins and impartial polysaccharides remain in solution under these conditions. In solutions of high ionic vigor, CTAB will not precipitate nucleic acids and forms complexes with proteins. CTAB is therefore useful for refining nucleic acid from organisms which produce large quantities of polysaccharides such as plants and certain gram-negative bacteria [9]. Valderrama-Chairez et al. used SDS buffer for the extraction of RNA from cactus fruit and concluded that the quality was high, but showed less intensity with DNA contamination compared to the CTAB methods. While, reported that the RNA yield using CTAB method was higher in plants compared to the yield using SDS buffer [12].

**Fig. 2:**
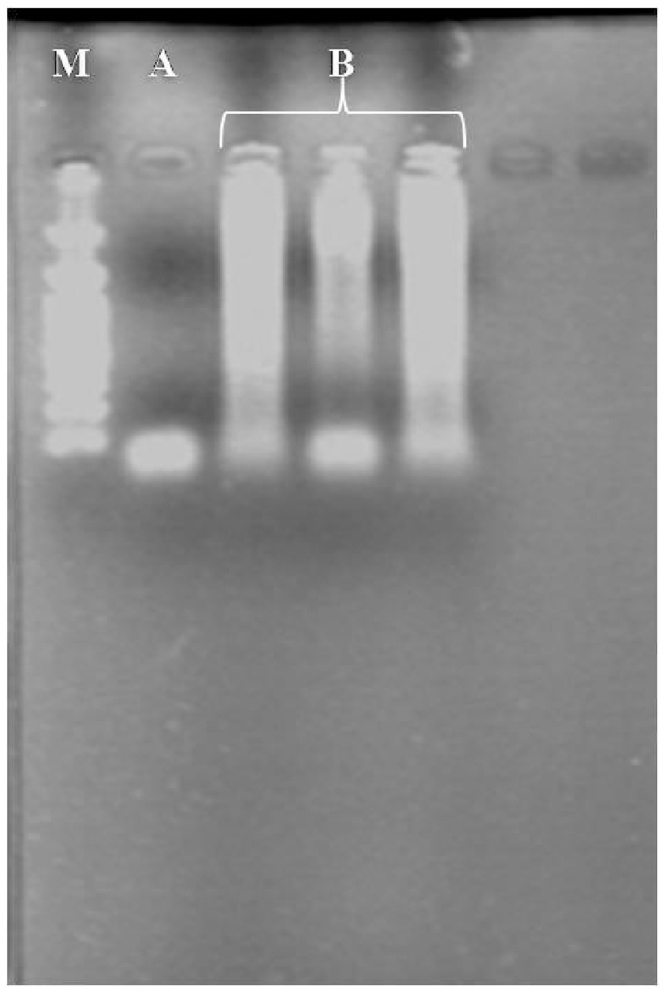
Agarose gel electrophoretic analysis of RT-qPCR amplified cDNA of ACTIN specific primers. M: molecular marker (100 bp Ladder), A: RNA control and B: PCR products after 40 cycles with ACTIN specific primers using cDNA generated from total RNA isolated as template.

Finally, the quality of RNA was evaluated by cDNA synthesis using the RT-qPCR reaction, and the level of expression of the gene of actin and other genes were calculated in this study. The cDNA was successfully displayed with good yield and reverse transcription products, indicated clear bands in 1% agarose/TBE gel electrophoresis (Fig. 2). These results indicated that the total RNA obtained had a sufficient quality for using in RT-qPCR analysis and molecular studies. This is the first report of high-quality RNA extraction from kiwifruit.

In the present study, the results of the new protocol with the results of other protocols that were designed to extract RNA from plant tissues containing high polysaccharides and polyphenols have been compared (Table 3). Hu et al. had described the simple method of CTAB buffer with 1.4 M of NaCl to extract RNA from kiwifruit that produced pure product with low yield (23 μg/g FW) which will eventually limit the success for downstream application such as PCR [13]. Conversely, Kumara and Costa had described an efficient protocol for RNA extraction of CTAB buffer with 1.4 M of NaCl in peel tissue of different banana cultivars which had an average yield (108.36–242.62 μg/g FW) with relatively low purity (near 2.0) [8]. Also, Djami-Tchatchou and Straker described a CTAB-based RNA extraction protocol and obtained 86.83 μg/g FW RNA and 164.67 μg/g FW RNA from the flesh and skin of *Persea americana* Mill., respectively, with high RNA purity [14]. While, our new protocol showed higher yield of RNA (∼2180.7 ng/μl or ∼545.175 μg/g FW) and higher purity from kiwifruit compared to the previous mentioned protocols. Wong et al. used high-salt CTAB buffer containing 4 M of NaCl in their protocol and resulted that the purity of RNA as well as the yield was low (164 ng/μL) from the stem of *Hylocereus sp* [11]. Also, Rezadoost et al. used CTAB buffer containing 1.4 M of NaCl in their protocol and resulted that the purity of RNA as well as the yield was low (258–364 ng/μL) from the leaf and root of *Betula pendula* and *Vitis vinifera* [15]. In our protocol, to inhibit the solubilization of polysaccharides in the RNA and DNA extract 1.4 M of NaCl salt concentration was used in the extraction buffer to precipitate RNA and DNA and the problem of polysaccharide contamination was solved. At this level, the polysaccharides remained in the solution and were disposed with ethanol supernatant to reduce the levels of polysaccharides. The procedure described here is quick and simple enabling the processing of a large number of samples easily.

**Table 3:** Yield and purity of RNA extract from tissues containing high polyphenols and polysaccharides compounds using different methods.

Protocol	Average RNA purity (A_260_/A_280_)	Average RNA purity (A_260_/A_230_)	Average RNA yield (μg/g FW)	Tissues
The new protocol	2.07	2.43	545.175	Kiwifruit
Hu et al. [13]	1.92	1.62	23	Kiwifruit
Kumara and Costa [8]	1.65–2.01	1.68–2.03	108.36–242.62	Banana (*Musa* spp.)
Djami-Tchatchou and Straker [14]	2.14	2.27	86.83–164.67	*Persea americana Mill.*
Wong et al. [11]	1.57	–	64 (ng/μl)	*Hylocereus* sp.
Rezadoost et al. [15]	2.04	2.01	285–364 (ng/μl)	*Betula pendula* and *Vitis vinifera*

## Additional information

Kiwifruit, *Actinidia deliciosa* has a large amount of carbohydrates, polyphenols, proteins, minerals and vitamin C. About 50% of the soluble protein content of kiwifruit is actinidin, an enzyme classified in the group of cysteine proteases. The proteolytic activity of actinidin is equivalent but, not similar to papain in papaya, ficin in fig and bromelain in pineapple. In plants, proteases, particularly cysteine protease, have different roles including: deposition of storage proteins in developing seeds, degradation of storage proteins during germination and seedling growth and biotic stresses [16,17].

High quality RNA extraction is an important step for gene expression studies. As a nucleic acid, RNA is used for protein synthesis, also broadly used in investigation of gene expression pattern in different plants. Type and quantity of RNA in plants are depending on the expression of special genes, which leads to a special phenotype. Earning adequate quantity of pure RNA is more challenging for quantitative Real Time PCR (qPCR) analysis that become difficult because of the contaminants presence such as proteins, polyphenols, polysaccharides and secondary metabolites [[15], [16], [17], [18]].

RNA and DNA extraction from kiwifruit are very difficult even with using the best kits. The extraction of RNA and DNA is based on the CTAB method including β-mercaptoethanol and PVPP using Phenol/Chloroform/isoamyl alcohol (25:24:1) to remove protein and polyphenols, followed by LiCl and sodium acetate to eliminate polysaccharides. According to the current results, the protocol 1 developed in this study allowed high yield and quality of RNA and DNA isolation from kiwifruit. This new protocol gave a better absorption ratios and RNA yields than the previous protocols. The high absorption ratios indicate a very low polyphenols, polysaccharides and proteins contaminations. This protocol can be applied for all fleshy fruits containing high polyphenols and polysaccharide compounds for further RT-PCR analysis and molecular studies. Also, this protocol is rapid and efficient, and can be completed successfully in a period of approximately 4 h.

## Conflicts of interest

The authors declare no conflicts of interest.
